# Observations on carapace color change in the juvenile big-headed turtle (*Platysternon megacephalum*)

**DOI:** 10.7717/peerj.7331

**Published:** 2019-07-26

**Authors:** Dainan Cao, Yan Ge, Yufeng Wei, Haoran Duan, Shiping Gong

**Affiliations:** Guangdong Key Laboratory of Animal Conservation and Resource Utilization, Guangdong Public Laboratory of Wild Animal Conservation and Utilization, Guangdong Institute of Applied Biological Resources, Guangzhou, China

**Keywords:** Big-headed turtle (*Platysternon megacephalum*), Carapace color change, Body coloration, Melanin deposition

## Abstract

The carapace color of newborn big-headed turtles (*Platysternon megacephalum*) is polymorphic and usually consists of two phenotypes: yellowish brown and olive green. As the turtles grew, over the first year of life, its carapace gradually turned from yellowish brown to chestnut brown, or from olive green to dark brown, depending on the phenotype. Meanwhile, the turtle’s plastron remained an orange and black pattern and did not change much. In this study, we primarily used HE staining to observe the carapace color change with age in big-headed turtle juveniles. We took the carapace marginal scute tissues twice from the same turtles before and after the carapace color change. Histological observations show that in the marginal scutes of the four tested turtles with different carapace color phenotypes, melanin granules are all concentrated in the dermal layer underneath the dorsal corneous layer, but rarely on the ventral side. Melanin deposits in the dorsal corneous layer were found to increase as the corneous layers thickened, while the melanin deposits in the ventral corneous layer did not change significantly. However, there was no significant difference in melanin deposition in the epidermis and dermis of the carapace among the yellowish brown, chestnut brown, olive green, and dark brown big-headed turtles. The results of our study indicate that the carapace color darkening in big-headed turtles may not be due to changes in melanin content of the carapace, but is the result of melanin accumulation and superposition in the dorsal corneous layer.

## Introduction

Body coloration is one of the major morphological features of animals and can change in response to factors such as temperature, light, habitat characteristics, and the vision and behavior of prey and predators (Roulin, 2004). Body coloration change is a common way for animals to adapt to the natural environment and plays an important role in self-protection and mate selection. There are two principal types of color change: (i) short-term physiological color change, such as in some fish (Rhodes & Schlupp, 2012) and chameleons (Teyssier et al., 2015), where body color can be adjusted in minutes or seconds; and (ii) long-term morphological color change, where changes in the skin color of some animals may take days or months (Ichikawa, Ohtani & Miura, 1998; Leclercq, Taylor & Migaud, 2010).

In turtles, the color pattern of the shell may be permanent or may gradually change with age or environment, in a process usually taking several months (Cao et al., 2018; Rowe et al., 2014). The substrate can induce melanization in seven turtle species (*Graptemys geographica*, *G. ouachitensis ouachitensis*, *Trachemys scripta elegans*, *Chelydra serpentina serpentina*, *Sternotherus odoratus*, *Apalone spinifera*, and *A. mutica*): after rearing individuals on black or white substrates for 160 days, turtles maintained on a white substrate were generally lighter while turtles maintained on a black substrate were generally darker (Rowe et al., 2014). Increasing the amount of carotenoids in the food can increase the yellow chroma in chin stripes and increase red chroma in the neck and carapace stripes on painted turtles (Steffen, Hultberg & Drozda, 2019).

**Figure 1:**
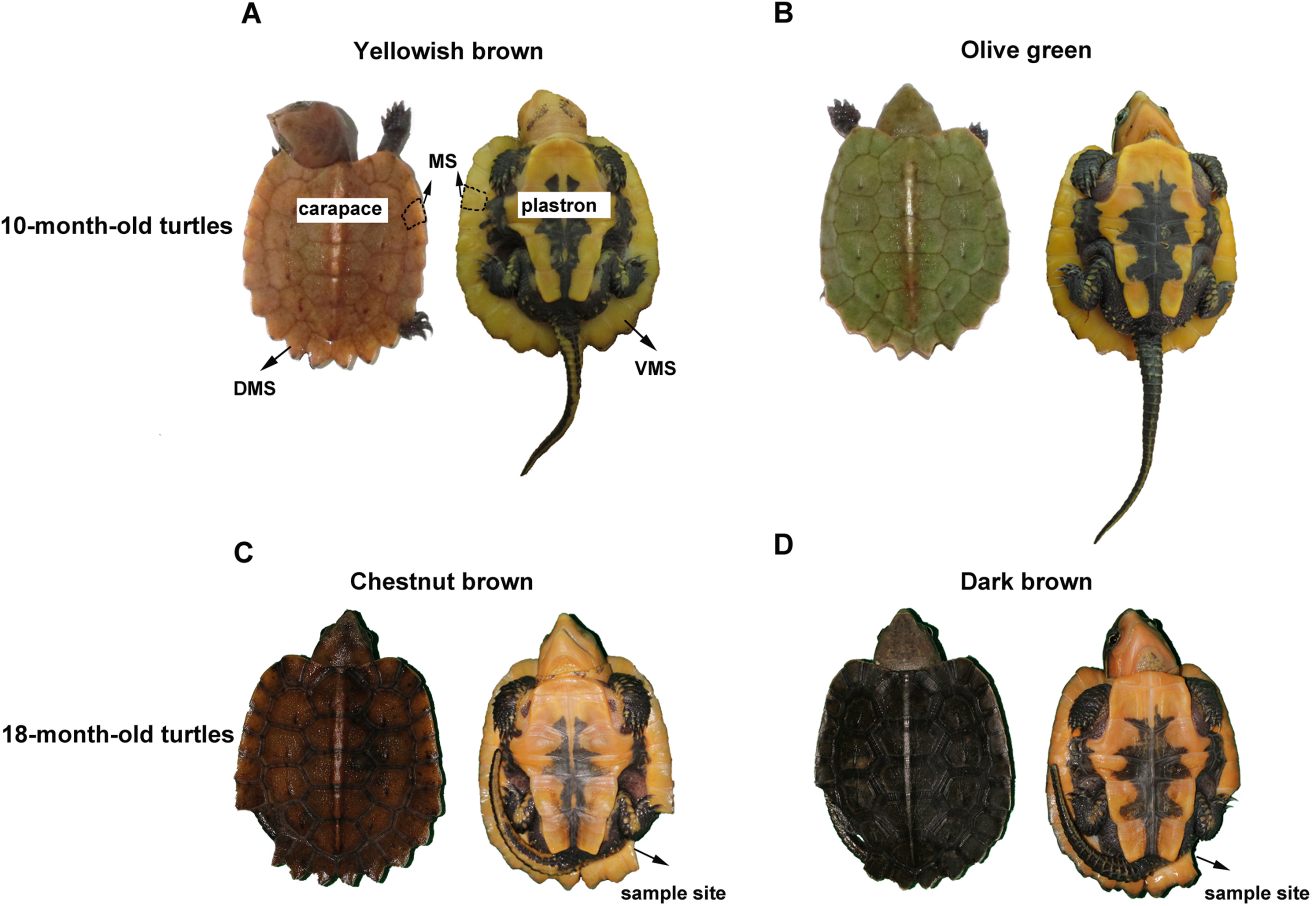
The carapace color change of the big-headed turtle. (A) shows a 10-month-old yellowish brown turtle. The carapace and plastron were marked at the corresponding positions and the part enclosed by the dotted line was a piece of marginal scute on the carapace. (B) shows a 10-month-old olive green turtle. (C) shows the turtle shown in (A), whose carapace color has changed to chestnut brown by the age of 18 months. (D) Is the turtle shown in (B), whose carapace color has changed to dark brown by the age of 18 months. The missing portions of the marginal scute shown in (C) and (D) were the sample sites. MS, marginal scute; DMS, the dorsal side of the marginal scute; VMS, the ventral side of the marginal scute.

The big-headed turtle, *Platysternon megacephalum*, inhabits rocky mountain streams in southeast Asia (Rhodin et al., 2017). In general, the carapace of the newborn big-headed turtle appears yellowish brown or olive green, while the plastron has an orange and black pattern (Figs. 1A and 1B). As the turtle grows, the carapace color gradually darkens. At about one and a half years old, the yellowish brown has become a chestnut brown (Fig. 1C), while the olive green turns to a dark brown (Fig. 1D). Over the same time period, changes in the color and pattern of the plastron are not obvious. Thus, the marginal scutes of the carapace are special: their dorsal side changes color like the other scutes of the carapace, while the ventral side remains orange (Fig. 1).

In this study, we primarily used HE staining to investigate the carapace color change with age in big-headed turtle juveniles. The marginal scute sections were taken twice from the same turtles before and after the carapace color change. We observed the distribution of melanin in the marginal scutes of different color phenotype turtles to see if the melanin deposition in the darker color turtle carapaces was different from that of the lighter color turtle carapaces, and whether the melanin deposition in the dorsal and ventral side of the marginal scutes was different. Given that the change in color of the two phenotypes is due to melanin deposition, the darkening of yellowish brown to chestnut brown as well as olive green to dark brown is predicted to be the result of changes in melanin deposition and melanin accumulation.

## Materials and Methods

### Sample collection

Five yellowish brown and five olive green big-headed turtles (10 months old) were chosen from the Xiangtoushan National Nature Reserve in Huizhou, Guangdong, China. Experiments were conducted in accordance with the Guide for the Care and Use of Laboratory Animals (Council, 2010) and were reviewed and approved by the Animal Ethics Committee at the Guangdong Institute of Applied Biological Resources (GIABR-AE-2017001). A small piece of carapace marginal scute measuring less than 0.25 cm^2^ was biopsied from each turtle for HE (hematoxylin and eosin) staining. The sampling sites are shown in Fig. 1. Then all the sampled turtles were reared under the same conditions untill 18 months old, when their carapace turns from yellowish brown to chestnut brown, or from olive green to dark brown, respectively. All the 10 color-changed big-headed turtles were sampled again in the same way at 18 months old, with the sampling position next to the first sampling site on the carapace marginal scute.

### HE staining

The collected carapace samples were fixed in 4% paraformaldehyde, and embedded in paraffin. Sagittal sections of six to eight μm thickness were stained with hematoxylin-eosin and then prepared for light microscopy. Images were captured using a Nikon Eclipse 80i microscope (Nikon, Tokyo, Japan).

### Melanin deposition analysis

Adobe Photoshop CS3 (Adobe Systems Inc., San Jose, CA, USA) was used to detect the melanin deposition. In the HE staining image of each carapace marginal scute tissue, we took three equal sized, non-overlapping image cuttings covering as near the same zone in terms of organizational structure. The cropped HE staining image was opened in a new window, and the pixels of the total HE staining image was collected using the histogram tool. The melanin deposition sites were selected and the pixels calculated the same way. The melanin deposition ratio (MR) was calculated using the formula MR = SP/TP, where SP = pixels of selected melanin deposition sites region and TP = pixels of the total HE staining image. GraphPad Prism 7 (GraphPad Software Inc., San Diego, CA, USA) was used to analyze the pixel value obtained from Photoshop.

### Statistical analysis

Each experiment was independently repeated three times. All data was expressed as means ± S.D. and analyzed by ANOVA using the GraphPad Prism 7. For all analyses, a *P*-value < 0.05 was regarded as statistically significant.

## Results

### Histological observations on carapace color change in the big-headed turtle

**Figure 2:**
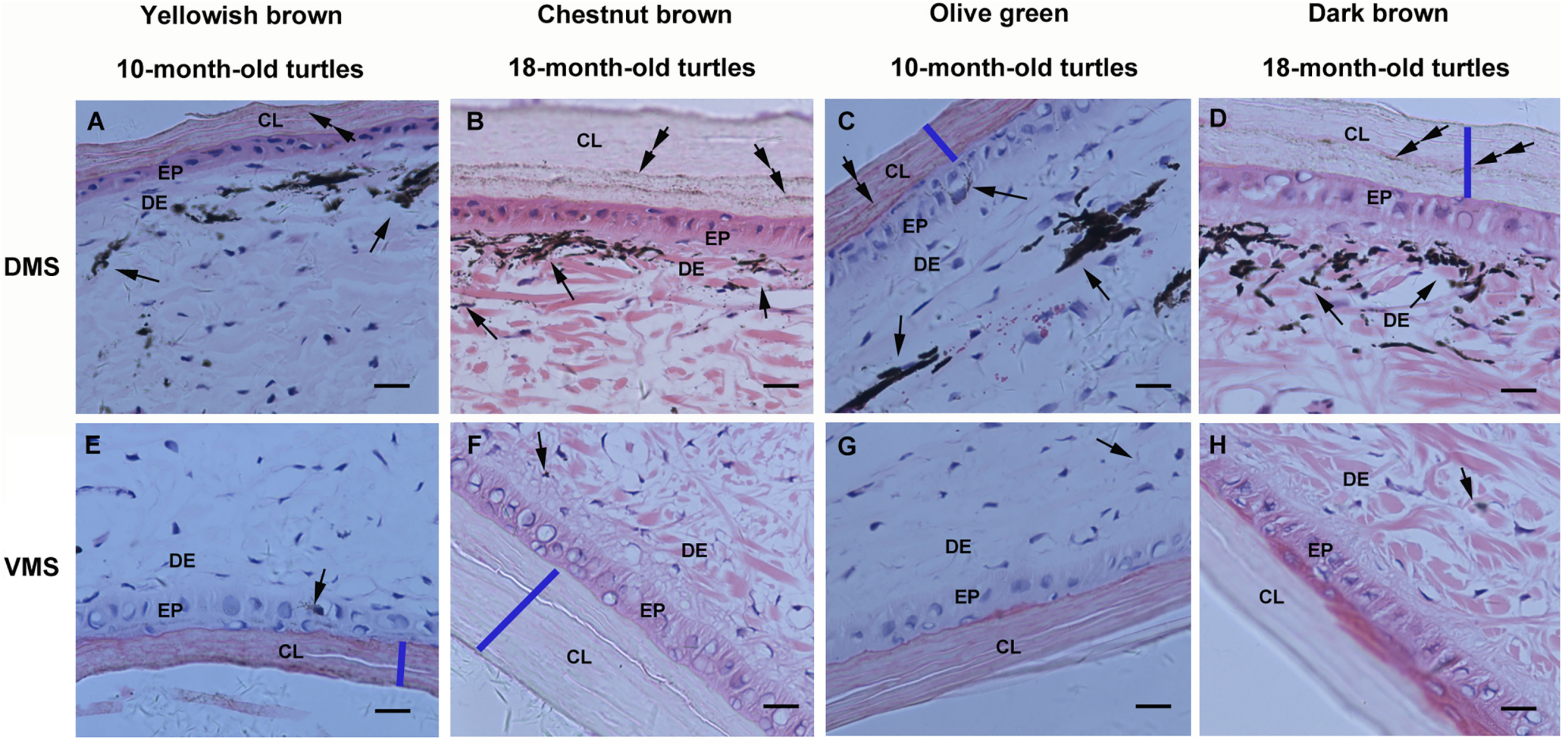
Light micrographs of the marginal scute of carapaces from yellowish brown, chestnut brown, olive green, and dark brown big-headed turtles, stained with hematoxylin and eosin. On the dorsal side (A–D) of the marginal scute from each different color phenotype turtle, melanin granules (arrows) were visible underneath the corneous layer and melanin deposits (double arrows) were observed in the corneous layer, whereas they could hardly be detected on the ventral side (E–H). The bule lines were drawn to mark the thickness of the corneous layer. CL, corneous layer; EP, epidermis; DE, dermis; DMS, the dorsal side of the marginal scute; VMS, the ventral side of the marginal scute; Scale bars, 10 μm.

Structural observations on the marginal scute of the big-headed turtle showed that it is a sandwich-like structure with two exterior corneous layers and an interior layer, with epidermal layers underneath the corneous layers and dermis underneath the epidermis (Fig. S1). Histological observations of the marginal scute from yellowish brown, chestnut brown, olive green, and dark brown big-headed turtles consistently showed that melanin granules were concentrated in the dermis underneath the corneous layer on the dorsal side (Figs. 2A–2D), but rarely on the ventral side (Figs. 2E–2H). By comparing marginal scute slices of 10-month-old turtles and 18-month-old color-changed turtles, it was observed that the two exterior corneous layers of the carapace grew thicker increased with age (the thickness of the corneous layers were indicated by blue lines). The melanin deposits in the dorsal corneous layer (double arrows) of 10-month-old turtles were less (Figs. 2A and 2C), while the melanin deposits in the dorsal corneous layer of the 18-month-old turtles increased (Figs. 2B and 2D), indicating that melanin accumulated into the dorsal corneous layer with age. In contrast, no increase in melanin deposition was observed in the ventral corneous layer of the older turtles (Figs. 2E–2H).

### Melanin deposition analysis

**Figure 3:**
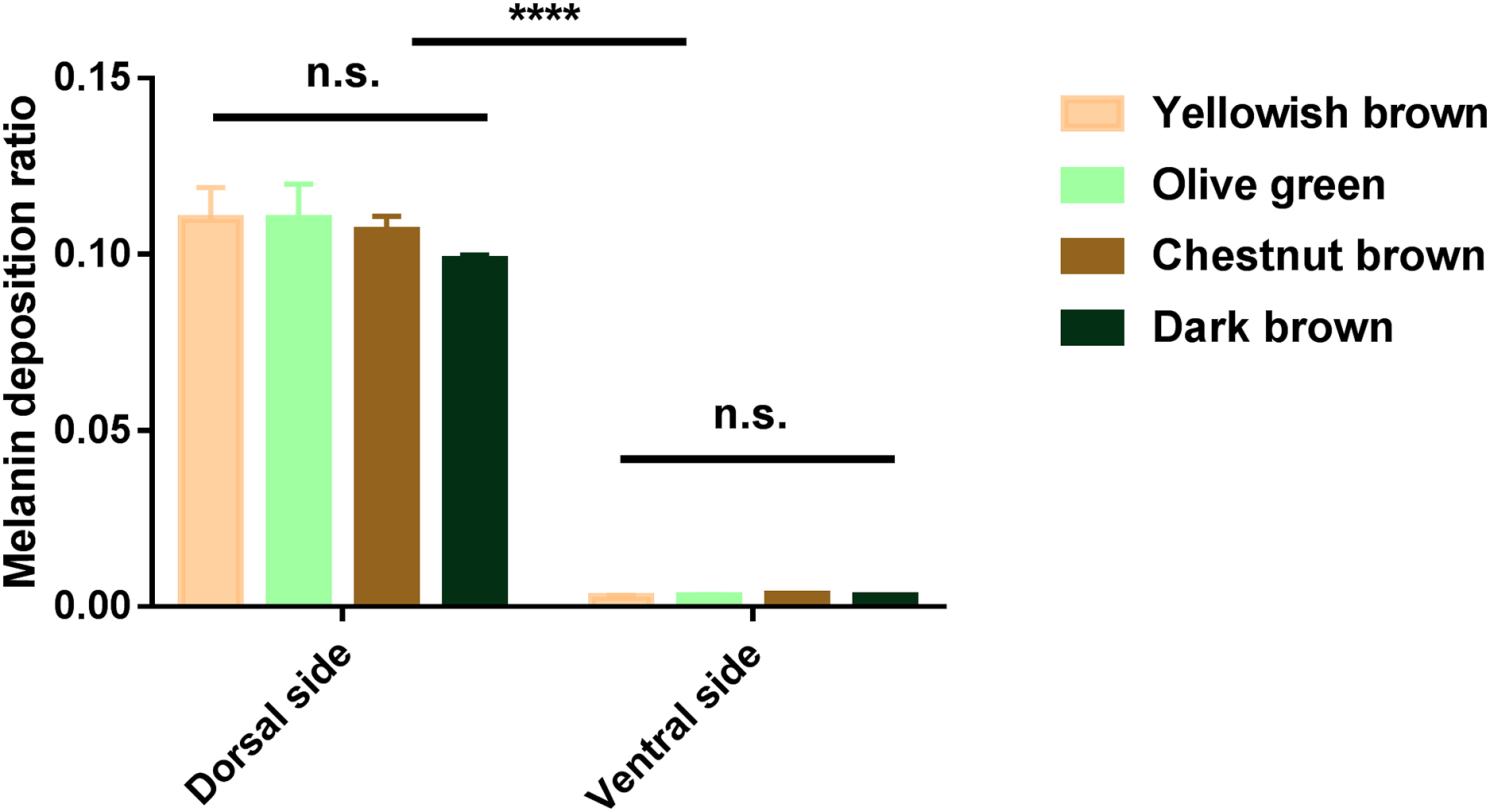
Analysis of melanin deposition ratio in marginal scutes of big-headed turtles with different carapace color phenotypes. There were no significant differences among different carapace color phenotypes in the dorsal or ventral sides of the marginal scutes. There were significant differences in melanin deposition between the dorsal and ventral sides of marginal scutes in all four phenotypes. Data are means ± S.D.; ****P < 0.0001; n.s., not significant.

In order to compare the relative ratio of melanin deposition in the younger yellowish brown and olive green phenotypes, as well as the older chestnut brown and dark brown carapace, the HE slices were saved as .TIFF images and the pixels of melanin spots were analyzed by Photshop. Among the turtles of different carapace color phenotypes, we found no significant differences in melanin deposition in the dorsal (*N* = 12, *F* = 1.819, *P* > 0.05) or ventral (*N* = 12, *F* = 1.596, *P* > 0.05) sides of their marginal scutes (Fig. 3). On the other hand, there was a significant difference in melanin deposition between the dorsal and ventral sides of marginal scutes in turtles of different phenotypes (*N* = 24, *F* = 2,082.048, *P* < 0.0001, Fig. 3).

## Discussion

The color and pattern diversity exhibited by the organism mainly depends on the content and distribution of various pigments, especially melanin, on the surface of the body (Aspengren, Sköld & Wallin, 2009). Pigments selectively absorb light at certain wavelengths while allowing light at other wavelengths to be reflected (Morehouse, Vukusic & Rutowski, 2007). Color changes can be produced through the changes in the number and concentration of pigment cells or dispersion or aggregation of pigment-containing organelles (Nilsson Sköld, Aspengren & Wallin, 2013). The other mechanism of color production is termed structural coloration and is produced by scattering, interference, and diffraction of light caused by nano-scale reflective tissues or by ridges, lines, particles, etc. on the surface of animals. The multilayer nano-reflectors with alternating high and low refractive indices could generate light wave interference, causing rapid changes in body color (DeMartini, Krogstad & Morse, 2013). Moreover, pigmentary and structural color mechanisms frequently interact in many cases (Stoddard & Prum, 2011).

Turtle shells occur in a variety of colors and patterns and can become lighter or darker over time or depending on the environment. In our previous research on carapace color change in juvenile red-eared sliders (*T. scripta elegans*), we found no significant difference in melanin deposition in the carapace among the olive green, yellow green, and yellow brown slider turtles (Cao et al., 2018). In red-eared sliders, the distribution of melanin was found on the dorsal and ventral sides of the turtle carapace marginal scutes of each phenotypes. However, in the big-headed turtle, the melanin granules present in the marginal scute of all the four tested phenotypes were concentrated in the dermal melanophores underneath the dorsal corneous layer, but rarely on the ventral side. This phenomenon indicates that the concentration of pigment cells in the dorsal dermis may be the origin of the dark pigmentation observed on the surface of the carapace while the light orange observed on the ventral surface may be due to the lack of melanin deposition.

As both the yellowish brown and olive green turtles grew older, their carapace became darker, the corneous layers thickened, and melanin deposits in the dorsal corneous layer increased, while the melanin deposits in the ventral corneous layer did not change significantly. A previous study has speculated that as turtles grew, the thickness of the corneous layer in the softer epidermis (neck and tail) increased and the size of scales in the carapace or plastron increased. During epidermal cell proliferation, epidermal melanocytes transferred melanosomes into newly formed keratinocytes (Alibardi, 2013). The increased melanin deposits observed in the thickened dorsal corneous layers of the animals in this study further corroborate this theory. The present results provide interesting new evidence that, in the marginal scute of the big-headed turtles, melanosomes are transferred in one direction and accumulated only in the dorsal corneous layer.

On the other hand, as indicated from the gross histological observations of the marginal scute, there was no significant difference in melanin deposition in dermal melanocytes among the yellowish brown, chestnut brown, olive green, and dark brown big-headed turtles. These results suggest that the carapace color change of the big-headed turtles may not be due to changes in melanin deposition in the carapace, but due to the melanin accumulation and superposition in the dorsal corneous layer.

## Conclusions

These results imply that in the carapace of big-headed turtles, melanonsomes are mostly produced by the dermal melanocytes underneath the dorsal corneus layer, and transferred one way into newly formed keratinocytes in the dorsal corneus layer. The accumulation and superposition of the melanin granules in the dorsal corneus layer causes the yellowish brown to gradually darken into chestnut brown and the olive green gradually darkens into a dark brown. The ventral surface, lacking melanin deposition, remains orange.

## Supplemental Information

10.7717/peerj.7331/supp-1 Supplemental Information 1 Microscopic view of the marginal scute of the carapace from a big-headed turtle. (A) shows the overall cross-section at the very edge of the marginal scute. (B) shows the cross-section of the internal region of the marginal scute. CL, corneous layer; EP, epidermis; DE, dermis; DMS, the dorsal side of the marginal scute; VMS, the ventral side of the marginal scute; Scale bars, 100 μm.

10.7717/peerj.7331/supp-2 Supplemental Information 2 The raw pixel ratios of the melanin dots in turtles of different carapace color phenotypes. Each data indicates the the ratio of the pixels of the melanin deposition area to the pixels of the total HE staining image.

## Additional Information and Declarations

### Data Availability

The following information was supplied regarding data availability: The raw pixel ratios of the melanin dots are available in Table S1. These data were used for statistical analysis to compare the relative ratio of melanin deposition in the younger yellowish brown and olive green, the older chestnut brown and dark brown turtle carapace, and to compare the melanin deposition in the dorsal and ventral side of the sides of marginal scutes in turtles of different phenotypes.
