# Supplementary figures and images for: Observations on carapace color change in the juvenile big-headed turtle (Platysternon megacephalum)
Source: PeerJ. 2019 Jul 26;7:e7331. doi: 10.7717/peerj.7331 (PMC6662560; doi:10.7717/peerj.7331)

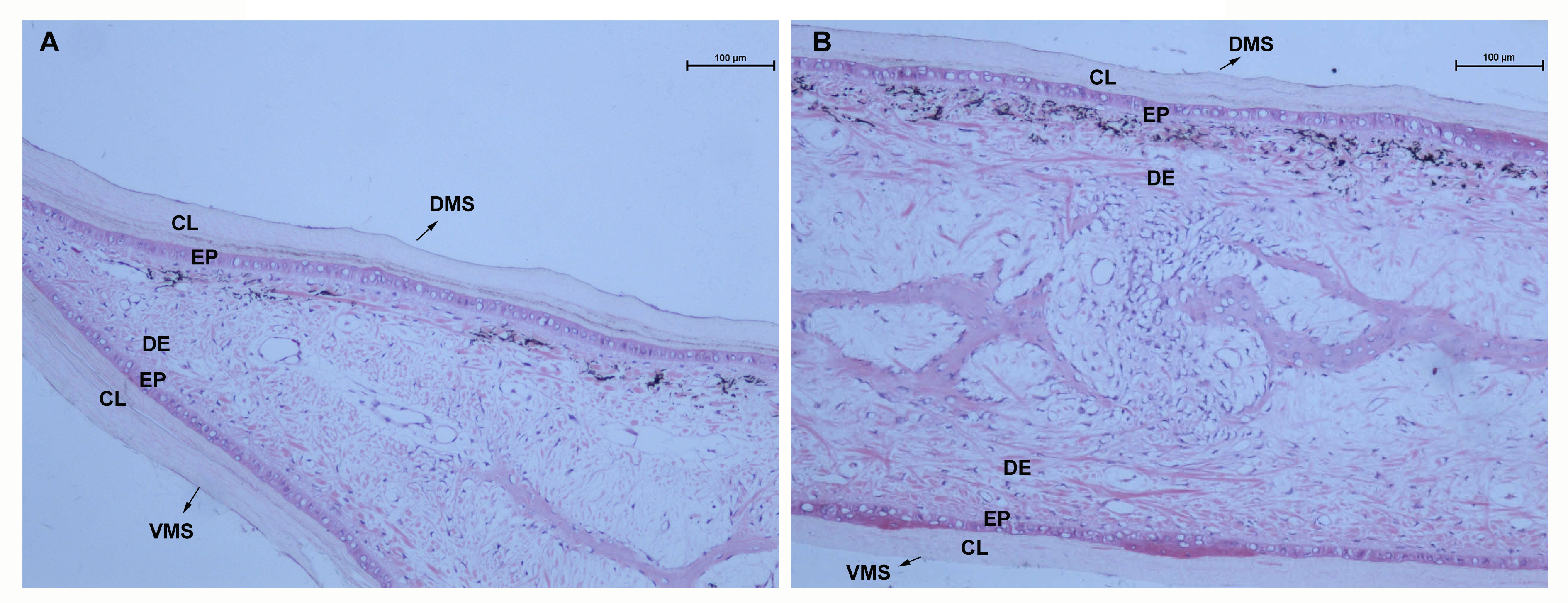

Supplement: Supplemental Information 1 — (A) shows the overall cross-section at the very edge of the marginal scute. (B) shows the cross-section of the internal region of the marginal scute. CL, corneous layer; EP, epidermis; DE, dermis; DMS, the dorsal side of the marginal scute; VMS, the ventral side of the marginal scute; Scale bars, 100 μm. [file peerj-07-7331-s001.png]
